# BRIDGING THE PRACTICE–RESEARCH DIVIDE: BUILDING SUCCESSFUL PARTNERSHIPS WITH STATE GOVERNMENT

**DOI:** 10.1093/geroni/igad104.1903

**Published:** 2023-12-21

**Authors:** Rajean Moone, Megan Hakanson, Tetyana Shippee

**Affiliations:** University of Minnesota, Minneapolis, Minnesota, United States; UMN School of Public Health, Woodbury, Minnesota, United States; University of Minnesota, Minneapolis, Minnesota, United States

## Abstract

In 2021 Minnesota implemented systemwide regulatory reform to transform loosely regulated housing with services establishments into licensed assisted living facilities. The new framework aligns assisted living facilities more closely with nursing homes. Today over 2,000 assisted living facilities are licensed in Minnesota ranging in size from two to 377 residents. While considered consensus legislation by consumer and provider groups, small, culturally specific providers were largely absent from negotiations. This absence has resulted in disparities in conforming to new regulatory requirements. The University of Minnesota has partnered with the Minnesota Department of Health to conduct analyses of regulatory inspection surveys. The analysis includes comparisons between small (under 15 residents) and large (over 15 residents) facilities as well as qualitative interviews of licensed directors of assisted living facilities that serve culturally specific communities. This paper focuses on the nature of the partnership between the University and state to organize and obtain survey inspection results and the subsequent development of recommendations to enhance regulatory policy.

